# A novel approach to enhance ACL injury prevention programs

**DOI:** 10.1186/s40634-018-0137-5

**Published:** 2018-06-18

**Authors:** Alli Gokeler, Romain Seil, Gino Kerkhoffs, Evert Verhagen

**Affiliations:** 1 Luxembourg Institute of Research for Orthopedics, Medicine and Science in Sports, 76 rue d’Eich, L-1460 Luxembourg, Luxembourg; 2 Department of Applied Neuroscience in Sports and Exercise, Institute of Sports Medicine, University of Paderborn, Paderborn, Germany; 3 University of Groningen, University Medical Center Groningen, Center for Human Movement Sciences, Groningen, Netherlands; 4 Academic Center for Evidence based Sports Medicine (ACES), Academic Medical Center, Amsterdam, Netherlands; 5 Department of Orthopaedic Surgery, Academic Medical Center, Amsterdam, Netherlands; 6 Amsterdam Collaboration for Health & Safety in Sports (ACHSS), IOC Research Center, Academic Medical Center/VU Medical Center, Amsterdam, Netherlands; 7 Department of Public and Occupational Health, Amsterdam Movement Sciences, VU University Medical Center, Amsterdam, Netherlands; 8 Division of Exercise Science and Sports Medicine, Department of Human Biology, University of Cape Town, Cape Town, South Africa

**Keywords:** Injury prevention, ACL, Motor learning, Sports specific, Athletic level

## Abstract

Efficacy studies have demonstrated decreased anterior cruciate ligament (ACL) injury rates for athletes participating in injury prevention programs. Typically, ACL injury prevention programs entail a combination of plyometrics, strength training, agility and balance exercises. Unfortunately, improvements of movement patterns are not sustained over time. The reason may be related to the type of instructions given during training. Encouraging athletes to consciously control knee movements during exercises may not be optimal for the acquisition of complex motor skills as needed in complex sports environments. In the motor learning domain, these types of instructions are defined as an internal attentional focus. An internal focus, on one’s own movements results in a more conscious type of control that may hamper motor learning. It has been established in numerous studies that an external focus of attention facilitates motor learning more effectively due to the utilization of automatic motor control. Subsequently, the athlete has more recourses available to anticipate on situations on the field and take appropriate feed forward directed actions. The purpose of this manuscript was to present methods to optimize motor skill acquisition of athletes and elaborate on athletes’ behavior.

## Background

Despite the preventive efforts introduced over the past decades, anterior cruciate ligament (ACL) injury rates in sports have unfortunately not decreased (Agel et al., 2016). From 2004 through 2013, statistically significant increases in the average annual number of injuries (controlled for athletic exposures) have been reported for men’s and women’s college basketball, ice hockey, field hockey, football, and volleyball (Agel et al., 2016). The efficacy of ACL injury prevention programs appears to primarily affect young female athletes who can expect about a 52% reduction of ACL injury risk when commencing an ACL injury prevention program (Sadoghi et al., 2012). The literature is scarce regarding the efficacy of prevention programs to reduce ACL injuries in male athletes (Alentorn-Geli et al., 2014). A cluster randomized study revealed that male football players who were allocated to a FIFA11+ intervention (Soligard et al., 2008) group had a lower incidence of ACL injuries compared to those who followed their routine warm-up (Silvers-Granelli et al., 2017). It should be noted however, that reduction of ACL injuries was only achieved in players in the lower divisions but not those who played in the higher divisions (Silvers-Granelli et al., 2017). In other words, efficacy of current ACL injury prevention programs has not been demonstrated for all age groups of different sex, level of play and type of sports.

Based on the aforementioned, there is room and need for optimization of current ACL injury prevention programs. To present an overview, this commentary has been outlined in four sections. First, it analyzes the current knowledge of ACL injury mechanisms. In the second section we will review risk factors for ACL injuries. Thirdly, the content of existing ACL injury prevention programs will be discussed. Finally, we will present ACL injury prevention interventions based on principles of motor learning that aim to improve motor skills of the athlete in a context of an actual game.

### What is known about the non-contact ACL injury mechanism?

A non-contact ACL injury mechanism usually involves single-legged landing or sidestep cutting (Krosshaug et al., 2007). Slight player-to-player contact may also contribute to the onset of injury (Koga et al., 2010). Even if there is no direct contact to the knee, slight physical contact results in a sudden change of planned movements of the athlete. This puts the athlete at risk for an ACL injury as there is only a very limited time frame for corrective action.

Differences in non-contact ACL injuries sustained during ball handling and defensive action in high school sports have been studied (Monfort et al., 2015). In high-school basketball, increased lower extremity injury risk was observed for those players involved in defensive actions (Monfort et al., 2015). In football, no differences in injury rates were found between ball handling or defensive actions (Monfort et al., 2015). In a study that used video analysis of actual ACL injuries in high school, college and at the professional level, found that the majority of injuries occurred during offensive actions (Krosshaug et al., 2007). The attention of the injured player was most commonly focused at the basket rim, followed by an opponent or a focus on the ball (Krosshaug et al., 2007). Walden et al. identified a top three of non-contact ACL injury mechanisms in professional football which were 1) pressing with a defensive action towards opponent, 2) re-gaining balance after kicking and 3) landing after heading (Walden et al., 2015). Similar for youth female and male and elite female handball, the majority of ACL and other lower extremity injuries occurred in the attacking phase by back or wing players doing a plant-and-cut maneuver or a single-leg landing movement (Olsen et al., 2004, Olsen et al., 2006). In elite female handball, some form of perturbation occurred, leading up to an ACL injury in 12 out of 20 cases (Olsen et al., 2004). These collective findings highlight differences in playing situations in various sports preceding an ACL injury.

Unfortunately, reflex mechanisms fail to protect the knee joint from injury, as an ACL injury occurs within 50 ms after ground contact (Koga et al., 2010), which is faster than the time needed by the central nervous system of about 120–140 ms to generate an appropriate response (Hopkins et al., 2009).

### Individual risk factors for non-contact ACL injuries

**Fig. 1:**
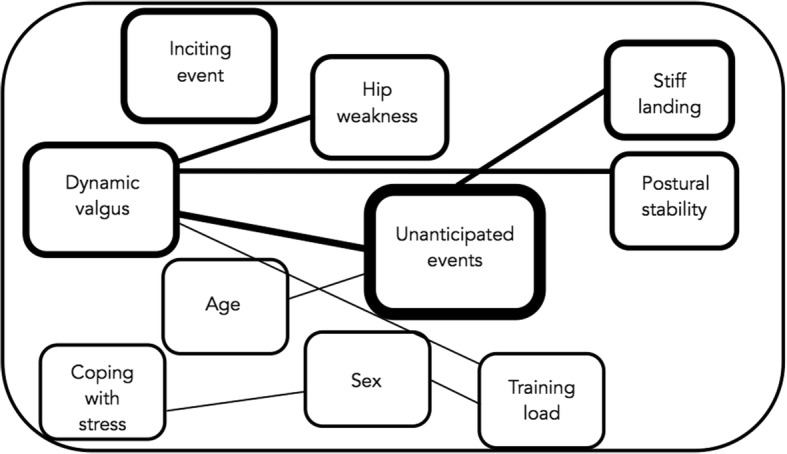
Complex model for sports injury (example 15 year old female football player). The interaction between the various risk factors are presented. The variables that represent risk factors circled by darker lines have more interactions than variables circled by lighter lines (adapted from Bittencourt et al. (Bittencourt et al., 2016)

ACL injury prevention programs are based on linear relationships between presence of risk factors and the actual occurrence of the ACL injury (Bahr, 2016). Recently, Bittencourt et al. (Bittencourt et al., 2016) proposed a complex system approach (Fig. 1) to enhance the understanding of injury etiology. Briefly, this approach highlights a non-linear interaction between risk factors from different dimensions (biomechanical, psychological, physiological and training characteristics) as a web of determinants, and how these may result in injuries (Bittencourt et al., 2016). One can appreciate the complexity of the interaction of various factors which may lead to an injury after an inciting event. Some of them are modifiable and are key components of current ACL injury prevention programs.

### Content ACL injury prevention programs: Structure and weaknesses

ACL injury prevention programs entail a combination of plyometrics, strength training, agility and balance exercises (Sadoghi et al., 2012, Gagnier et al., 2013). They are generally applied to entire teams without individualization to correct players’ specific deficiencies. The premise is that through such universal exercises, the athlete acquires sufficient neuromuscular control and strength to handle unexpected situations such as a sudden change of planned movements, that may result in high joint loads. Hence, the preventive effectiveness largely depends on neuromuscular feedback mechanisms which will be activated once the athlete encounters a potential injury mechanism.

ACL injury prevention programs typically include practicing pre-planned motor skills in a predictable environment with a focus on lower extremity alignment (Hewett et al., 2005). In the motor learning domain this is defined as practicing closed motor skills (Schmidt, 2005). One could argue that this approach lacks a transfer towards the unpredictable and complex demands placed on the athlete while on the field (Monfort et al., 2015). For example, in any injury mechanism, an athlete is embedded in a playing situation where external factors such as possession of a ball and position of team mates and opponents are involved (Olsen et al., 2004, Boden et al., 2009). These attentional and environmental interactions effects on neuromuscular function are largely not addressed in current ACL injury prevention programs (Grooms and Onate 2016). Ideally, athletes should acquire the ability to sustain optimal motor control while engaging in complex athletic environments, whilst minimizing their risk to sustain an injury.

### Potential ways of improvement

In ACL injury prevention programs, athletes learn motor skills in rather controlled conditions which rely on neuromuscular feedback mechanisms (Myklebust et al., 2003). Given the demands on the field, preventative training should focus on interventions that incorporate elements of anticipation, perturbations, focus of attention and visual-motor control within complex task environmental interaction (Grooms and Onate 2016). Adequate anticipation of a potential high-risk injury situation may give the athlete sufficient time to avoid the situation. In case the time frame is too short to avoid the situation, the athlete may have an opportunity to prepare for the change in direction and/or an upcoming perturbation. Such feed-forward mechanisms are important as it allows the muscles time to generate force and control correct lower extremity alignment during landing.

A potential limitation of current ACL injury prevention training is the lack of transfer from practiced exercises with high conscious control, to the automatic movements required for complex unanticipated events on the field (Benjaminse et al., 2015a, b, c). In the next section of this manuscript we will discuss various principles of motor learning that targets attentional and environmental factors. The goal is that athletes acquire the ability to sustain optimal motor control while engaging in complex athletic environments, whilst minimizing their risk to sustain an ACL injury. We acknowledge that this is only one piece of the puzzle amongst many other potential prevention strategies recently identified (Vriend et al., 2017).

### Principles of motor learning

#### Attentional focus

It is generally assumed that athletes benefit from information in the process of acquiring motor skills by directing the attention to movements (Beilock et al., 2002). Similarly, in ACL injury prevention, instructions direct the athlete’s attention to various aspects of movements. In the motor learning domain, this type of attentional focus is termed “internal focus” (Wulf et al., 1998). Instructions are directed towards the execution of the movements itself such as “keep the knee over the toe”; “land with a flexed knee”; “raise the knee to the level of the hip” or “land with your feet shoulder-width apart” (Risberg and Holm 2009, Wilk et al., 2012). Unfortunately, encouraging athletes to improve awareness and knee control during balance, cutting, jumping, and landing (Holm et al., 2004) requires attentional capacity. In turn, this limits the available capacity for fast and complex motor skills that are needed for quick responses to an opponent’s action.

**Fig. 2:**
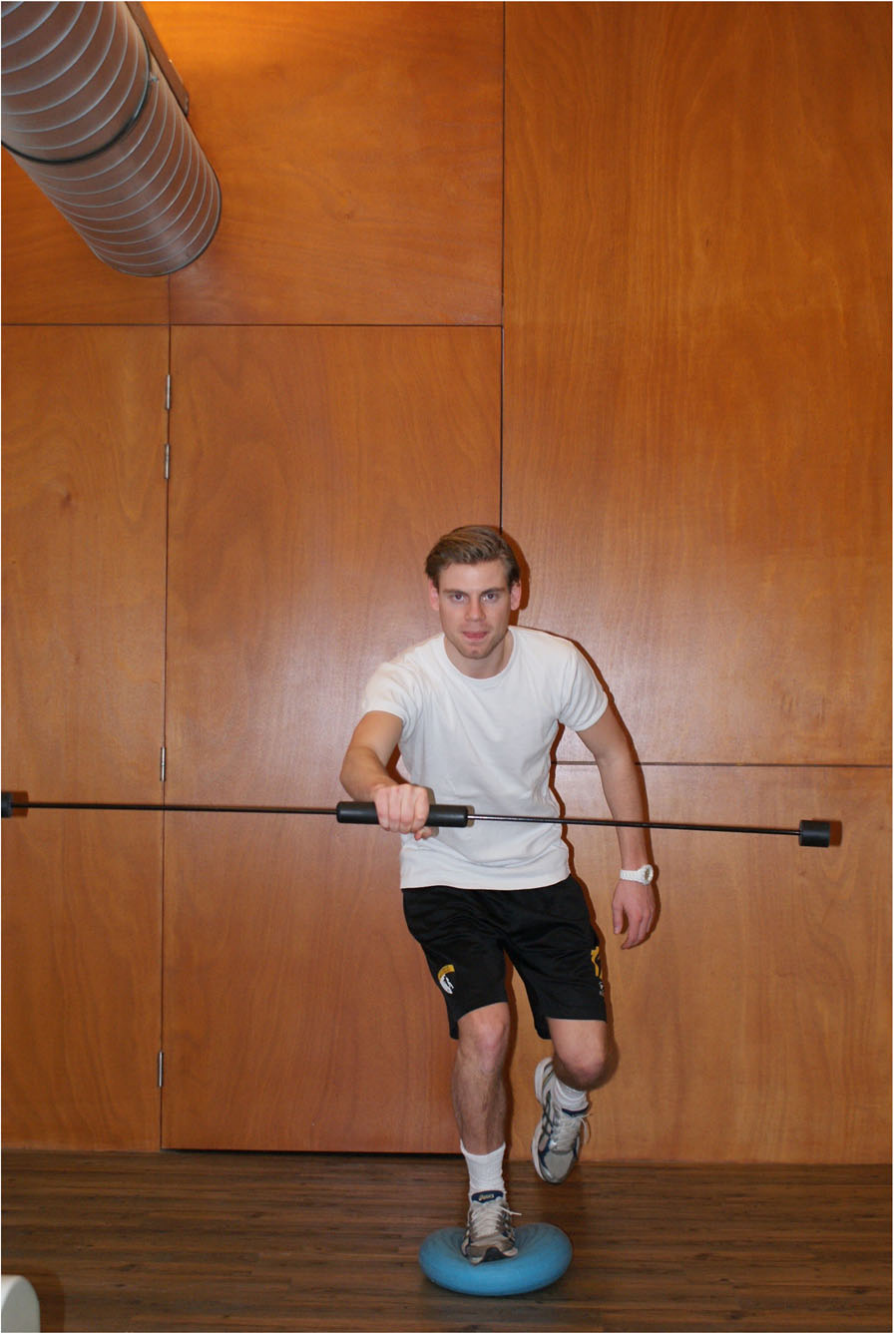
Postural stability. An athlete is practicing to improve postural stability. To promote an external focus of attention the athlete should be instructed to “focus on keeping the bar horizontal”. Instruction such as “stabilize your knee” are less effective because this induces an internal focus of attention

Conversely, an external focus of attention is induced when an athletes’ attention is directed towards the outcome or effects of the movement (e.g. landing from a jump: “try to land on the makers on the floor”). It has been established in numerous studies that an external focus of attention facilitates motor learning more effectively by utilization of unconscious or automatic processes (Wulf et al., 2001, Lohse et al., 2012, Lohse and Sherwood 2012, Wulf, 2012). Finding from a systematic review, clearly established that using instructions with an external focus result in better motor performance and movement technique (increased retention) compared to an internal focus of attention (Benjaminse et al., 2015a, b, c). This is illustrated by greater knee flexion angles, more center of mass displacement, lower peak vertical ground reaction force and improved neuromuscular coordination, while maintaining or improving performance (e.g. jump height, jump distance) (Benjaminse et al., 2015a, b, c). These findings are promising, as this yields an optimum between diminishing ACL injury risk (improved movement technique) without a reduction in performance. By using an external focus instruction, motor skills require less attentional demands as these are executed in a more automated fashion. Hence, more recourses are available to anticipate on situations on the field and take appropriate feed forward directed actions. An example is presented illustrating the use of an external focus of attention to improve postural stability (Fig. 2).

#### Implicit learning

The aim of implicit learning methods is to minimize the amount of explicit knowledge about movement execution that is accumulated during learning. One method is including “analogy” instructions during the acquisition of new skills. Analogy, or metaphoric description of the action, connects with a visual image, to help the athlete learn a movement skill (Liao and Masters 2001). For example, for an athlete to learn a softer landing strategy (more knee flexion) the instruction could be “when you land from a jump, try to image you’re landing on raw eggs and you don’t want to crack them”. The use of analogies may serve the same purpose by inducing an external focus of attention (Wulf and Lewthwaite 2016).

Moreover, one of the most interesting and widely unexplored aspects of implicit learning is its connection with factors of anticipation and decision making in relation to performance. It has been shown that expert athletes are better in these areas compared to less experienced athletes. They have an advantage in the speed and accuracy of their reactions, which is based on the ability to detect visual cues earlier and more precisely in the game’s patterns recognition and make better predictions of the opponent’s actions, even before some significant preparatory movements occur (Bishop et al., 2013).

A critical question is whether an athlete needs to be told what specific visual cues to look for, or can be learned without explicit verbal information (Farrow and Abernethy 2002). Implicit training using limited visual information about the direction of the ball in tennis, improved athletes’ prediction accuracy after the intervention. An explicit learning group, who received specific kinematic information about the tennis serve of the opponent, didn’t demonstrate any improvement in anticipatory skills (Farrow and Abernethy 2002).

A recent technological innovation has made it possible to modify visual input. These stroboscopic glasses (e.g. Senaptec Strobe, Senaptec, Beaverton, USA; Nike SPARQ Vapor Strobes, Nike Inc., Beaverton, USA) have the ability to partially obstruct vision by intermittently switching from clear to opaque, allowing highly complex, dynamic athletic maneuvers to be performed under degraded visual input (Grooms and Onate, 2016, Grooms et al., 2017). Interestingly, training with stroboscopic glasses has been shown to improve anticipatory skills (Smith and Mitroff 2012). Those athletes who trained with stroboscopic glasses achieved earlier and more accurate responses to visual cues compared to a control group (Smith and Mitroff 2012). Applied to ACL injury prevention, training athletes to improve their anticipatory skills may give them the much-needed window of opportunity to avoid high risk situations.

#### Video-feedback

Observational learning, as with video feedback is an effective way to enhance motor skill learning (Onate et al., 2005). In two randomized controlled trials (Benjaminse et al., 2015a, b, c, Welling et al., 2016), subjects received feedback during the two maneuvers in which ACL injuries most often occur: sidestep cutting and landing from a jump (Olsen et al., 2004). Recreational male basketball athletes who received visual feedback, were able to improve their sidestep cutting technique, whilst performance (running speed) was maintained compared over a group who received internal focus instructions (Benjaminse et al., 2015a, b, c). Similarly, in a double legged jumping task, video instruction had beneficial effects on landing technique in female and male athletes whilst performance (jump height) was maintained (Welling et al., 2016).

#### Differential learning

When using differential learning practicing movement skills, the movement patterns themselves are intentionally varied during practice. This principle suggests that by having athletes perform a variety of movement patterns, a self-organized process of learning is initiated (Schöllhorn et al., 2006). Through the process of experimentation with different movement patterns, target goals, and by learning alternative means of performing a task (rather than only practicing the supposedly correct movement form), athletes learn an individualized motor solution that works best for themselves given the environmental context and constraints of their own bodies (Magill and Hall 1990).

#### Contextual interference

The contextual interference in motor learning is defined as the interference in performance and learning that arises from practicing one task in the context of other tasks (Magill and Hall 1990). The amount of contextual interference may vary, between low contextual interference in blocked practice and random practice at the high end of contextual interference. Variability of practice (or varied practice) is an important component to contextual interference, as it places task variations within learning (Magill and Hall 1990). The variation as discussed here in context of contextual interference refers to the variation in planning of practice and is different than what Schmidt (Schmidt, 2005) proposed to practice with a lot of variation. Clinicians must decide how to best schedule practice to facilitate learning. Although varied practice may lead to poor performance throughout the acquisition phase, the variety of practice organization results in improved retention and transfer of motor learning (Porter and Magill 2010). Of note, skill level of an athlete is a factor that may need to be considered in terms of amount of contextual interference provided (Porter and Magill 2010). In general, lower level athletes benefit more from low contextual interference, whereas elite athletes do well with high levels of contextual interference.

### Practical implications and future directions

As outlined in the ACL injury mechanism section, the cascade of events taking place that led to the ACL injury are vastly different between sports and age groups. The results support implementing sport-specific interventions to account for the variation in playing situations. Subsequently future ACL injury prevention interventions should incorporate elements specific for type and level of sports tailored to the individual athlete. An athlete should be progressively exposed to comparable physical, environmental, and psychological stressors which they will face in the sport they participate in.

**Fig. 3:**
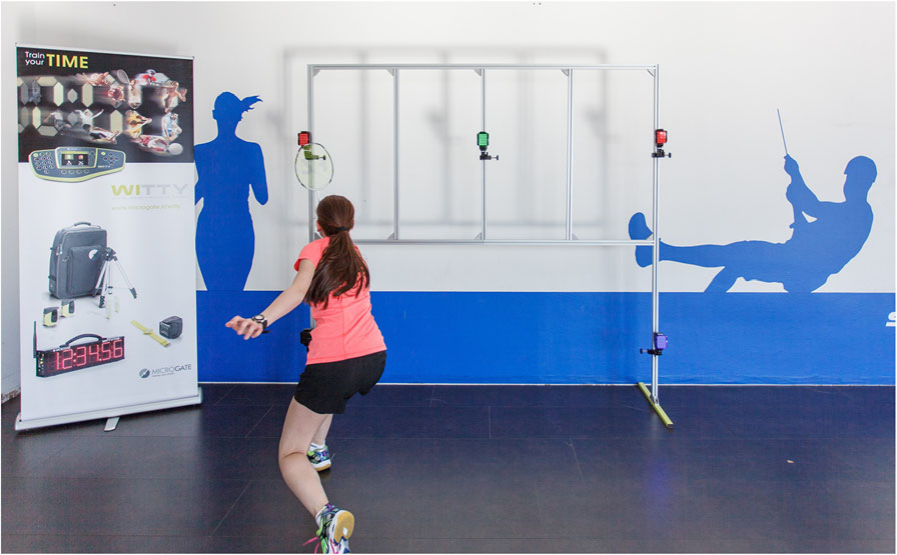
A badminton player practicing reaction task using the Microgate Witty SEM system (Microgate, Bolzano, Italy). The Microgate Witty SEM shown here consist of a framework holding three LED lights that illuminate green in a random order. In this set-up, the player has to respond quickly and move the racquet in front of the LED that turns green

An example is given how novel concepts may be incorporated in future ACL injury prevention (Fig. 3) that uses linking of attention, anticipation, decision making and reaction speed. Hence, reflecting a task-athlete-environment interaction that should match the context of the specifics and level of sports.

Based on the optimal challenge point framework, complexity and the related chance of achievement of the various skills should be adopted to the skill level of the athlete (Guadagnoli and Lee 2004). That means that elite athletes should have different levels of challenge compared to recreational athletes. Using principles of motor learning strategies in ACL injury prevention may enhance skill acquisition more efficiently and increase the transfer of improved motor skills to sports activities. This has been clearly established in various controlled studies, but needs to be validated in a real-world scenario. To increase evidence, future research should focus on which, if any, combinations of the presented novel techniques work best. The approach presented may also enhance adoption by athletes as novel motor learning based programs entail more specific elements they recognize from their sports.
